# Shared Gut Microbial and Functional Signatures Linking Parkinson’s Disease and Type 2 Diabetes Revealed by Function-Anchored Metagenomics

**DOI:** 10.3390/microorganisms13122705

**Published:** 2025-11-27

**Authors:** Ying Cui, Shiya Wang, Wenlu Zhao, Yitong Du, Lin Wang, Bingyu Han, Mingkai Zhang, Xiaojiao Xu, Sichen Wang, Xiaolong Ma, Xinran Xu, Yingying Zhao, Shuangjiang Liu, Yulin Wang, Houzhen Tuo

**Affiliations:** 1 Department of Neurology, Beijing Friendship Hospital, Capital Medical University, Beijing 100050, China; cuiying2030@163.com (Y.C.); m15553986917@163.com (S.W.); 13017688128@163.com (W.Z.); 18801118091@163.com (Y.D.); wanglin3057@163.com (L.W.); hanbingyu@bjmu.edu.cn (B.H.); zhangmingk0527@163.com (M.Z.); wsc13521915793@163.com (S.W.); mxl8397@163.com (X.M.); 18500094355@163.com (X.X.); zyytx@sina.com (Y.Z.); 2 Department of Neurology, Beijing Anzhen Hospital, Capital Medical University, Beijing 100013, China; 15554129590@163.com; 3 State Key Laboratory of Microbial Technology, Shandong University, Qingdao 266237, China; liusj@im.ac.cn

**Keywords:** Parkinson disease, type 2 diabetes mellitus, comorbidity mechanisms, gut microbiota, metabolic pathways, shotgun metagenomics

## Abstract

Parkinson’s disease (PD) and type 2 diabetes mellitus (T2DM) exhibit increasing comorbidity, yet the shared contribution of gut microbiota remains unclear. To investigate parallel microbial and functional alterations underlying PD, T2DM, and PD with diabetes (PDDM), we performed fecal metagenomic sequencing in 156 PD, 41 T2DM, and 44 PD with diabetes (PDDM) patients and 83 healthy controls (HC). PD and T2DM showed highly concordant microbial shifts, with 22 genera and 91 species consistently altered across disease groups compared with HC. Functional enrichment highlighted common perturbations in taurine and hypotaurine metabolism, retinol metabolism, the hypoxia-inducible factor-1 (HIF-1) pathway, and xenobiotic degradation, implicating disrupted oxidative stress responses, neuro-metabolic regulation, and detoxification. Key taxa, including *Limosilactobacillus fermentum*, *Lactobacillus porci*, and *Lactobacillus delbrueckii*, were increased and showed moderate positive correlations (|ρ| ≥ 0.3) with antioxidant/retinol–HIF-1, taurine–hypotaurine, and xenobiotic degradation pathways. *Bifidobacterium breve* (unadjusted analysis) was increased in PD and further enriched in PDDM, correlating with multiple beneficial pathways. *Bifidobacterium simiarum* (covariate-adjusted analyses) showed the broadest positive pathway associations, while selected *Bacteroides* species (e.g., *B. acidifaciens*) exhibited negative correlations with insulin-resistance pathways and positive correlations with steroid hormone biosynthesis. By contrast, *Butyricimonas vaginalis* showed negative correlations with HIF-1 and insulin signaling and with cytochrome P450-related drug metabolism. These findings provide the first systematic evidence of parallel taxonomic and functional dysbiosis in PD and T2DM, supporting gut microbiota as a shared mediator and potential therapeutic target in comorbidity.

## 1. Introduction

Parkinson’s disease (PD) is a progressive neurodegenerative disorder primarily characterized by motor impairment. Its prevalence is 0.1–0.2% among adults aged ≥ 18 years and 1.7% in individuals aged ≥ 65 years, making it a crucial public health burden that substantially reduces quality of life in older populations [1]. Diabetes mellitus (DM) is a metabolic disease defined by chronic hyperglycemia. Type 2 diabetes mellitus (T2DM) accounts for the vast majority of cases, and in this study DM refers specifically to T2DM. In 2021, the global prevalence of T2DM was 10.5%, and the number of affected individuals is projected to reach 783 million by 2045, ranking it among the fastest-growing global health challenges [2].

Multiple cohort studies and systematic reviews suggest an association between the two diseases. In an Asian cohort of 2,362,072 patients with T2DM followed for 6–9 years, the hazard ratio (HR) for incident PD ranged from 1.09 to 2.78, increasing with diabetes severity [3]. A cross-sectional study further reported higher odds of DM among 791 patients with PD compared with healthy individuals, with odds ratios (ORs) of 1.50 in men and 1.7 in women [4]. Pharmaco-epidemiological evidence also points to potential mechanistic links: longitudinal analysis indicates that use of dipeptidyl peptidase-4 (DPP-4) inhibitors and glucagon-like peptide-1 (GLP-1) receptor agonists is associated with reduced PD incidence (incidence rate ratios [IRRs] of 0.64 and 0.38, respectively) [5]. Other work suggests that levodopa use may be associated with a lower risk of DM among patients with PD [6]. Nevertheless, some studies did not observe significant associations between PD and DM [7]. Therefore, the interrelationship between the two diseases warrants investigation into features beyond clinical observations.

Hyperglycemia exerts broad, direct neurotoxic effects and activates mitogen-activated protein kinase (MAPK) pathways (e.g., p38 and JNK), collectively leading to impaired neural conduction and reduced axonal regeneration [8]. In addition, impaired neuronal insulin receptor (InsR) signaling disrupts dopaminergic transmission [9]. High-fat, obesity-promoting diets reduce the expression of InsR, the dopamine transporter (DAT), and the dopamine D2 receptor (D2R) in the putamen and caudate nucleus [10]. Both the substantia nigra and pancreatic β-cells are highly vulnerable to oxidative stress and toxic insults, resulting in decreased mitochondrial respiratory capacity and cell viability [11]. Further, pathological α-synuclein and islet amyloid polypeptide (IAPP) may mutually promote aggregation [12], and chronic inflammation contributes to the pathogenesis of both conditions [13]. Collectively, these observations support overlapping pathophysiological substrates linking PD and DM.

The gut microbiota—often regarded as the human “second genome”—has been increasingly implicated in both metabolic and neurological disorders [14,15]. Growing evidence indicates widespread dysbiosis in DM and PD, which may jointly contribute to disease pathogenesis by disrupting intestinal barrier integrity, altering microbially derived metabolites (e.g., short-chain fatty acids, trimethylamine N-oxide [TMAO], and tryptophan derivatives), and inducing immune dysregulation and chronic inflammation [16,17,18]. Modulating the gut microbiota has emerged as a promising therapeutic strategy to restore metabolic and neuroimmune homeostasis in both conditions [19]. Nevertheless, systematic comparisons are lacking to determine whether DM and PD share common microbial signatures and whether these convergent taxa mediate overlapping pathogenic mechanisms. Clarifying shared microbial community patterns and the potential mechanistic roles of dysbiosis-associated taxa in both diseases is therefore of substantial significance.

In this study, we applied comparative shotgun metagenomics to identify key taxa exhibiting concordant abundance shifts across PD, DM, and comorbid PD with DM (PDDM), alongside HC. By integrating functional pathway enrichment with taxon–function correlation analyses, we delineated shared features related to metabolic rewiring, oxidative stress responses, and xenobiotic clearance in both conditions. Furthermore, by comparing species-level profiles between PDDM and PD, we explored whether reductions in *Bacteroides*-derived species may contribute to the transition from PD to the comorbid state. Collectively, this design clarifies common comorbidity mechanisms and provides a theoretical basis for microbiota-targeted interventions.

## 2. Materials and Methods

### 2.1. Participants

From March 2022 to December 2024, we consecutively enrolled outpatients with PD from the Department of Neurology at Beijing Friendship Hospital. Based on the presence of type 2 diabetes (T2DM, hereafter “DM” in this study), participants were assigned to a PD group (PD, *n* = 156) or a PD with comorbid T2DM group (PDDM, *n* = 44). In addition, age- and sex-matched healthy controls (HC, *n* = 83) and patients with T2DM without PD (DM, *n* = 41) were recruited.

Eligible participants were 40–80 years old, of either sex. PD cases met the 2015 Movement Disorder Society (MDS) clinical diagnostic criteria. The diagnosis of T2DM was based on the American Diabetes Association criteria: fasting plasma glucose ≥ 7.0 mmol/L, or 2 h plasma glucose ≥ 11.1 mmol/L during an oral glucose tolerance test, or current use of antidiabetic medication. HC had no history of PD, DM, or other significant neurological diseases.

Exclusion criteria were active infectious diseases, malignancy, rheumatic disease, severe cardiac, pulmonary, hepatic, or renal insufficiency; acute or chronic gastrointestinal disease; and use of antibiotics, probiotics, or prebiotics within 60 days prior to enrollment.

The study involving human participants was reviewed and approved by the Ethics Committee of Beijing Friendship Hospital, Capital Medical University (approval No. 2022-P2-052; approval date: 24 March 2022). All procedures were conducted in accordance with the principles of the Declaration of Helsinki (1975; revised in 2013). Written informed consent was obtained from all participants prior to sample collection and data use.

### 2.2. Clinical and Lifestyle Data

We recorded sex, age, BMI, fasting blood glucose (FBG), disease duration, medical history, and administered standardized assessments, including the Unified Parkinson’s Disease Rating Scale (UPDRS)/UPDRS-III, Hoehn–Yahr staging, and a lifestyle questionnaire (dietary frequencies for vegetables and meats, smoking, alcohol, coffee), as well as the use of levodopa and MAO-B inhibitors (MAOBIs).

### 2.3. Stool Collection

Each participant provided at least one stool sample (>0.5 g) in a standardized, sealed tube containing a DNA stabilizer. Samples were transferred to −80 °C within 48 h of collection and stored until processing.

### 2.4. DNA Extraction and Shotgun Metagenomic Sequencing

Genomic DNA was extracted from stool using the DNeasy PowerSoil Pro Kit (250) (QIAGEN, Shanghai, China) following the manufacturer’s protocol. DNA concentration was measured with a Qubit^®^ 3.0 fluorometer (Life Technologies, Carlsbad, CA, USA), and integrity was assessed by 1% agarose gel electrophoresis.

Libraries were prepared with the Watchmaker enzymatic fragmentation DNA library preparation kit (Watchmaker Genomics, Boulder, CO, USA; cat. no. 7K0019-096), using 200 ng input DNA per sample. DNA was enzymatically fragmented to an average insert size of ~350 bp. Libraries were first quantified on a Qubit 3.0, and fragment size distributions were verified on a fragment analyzer. Library concentrations were then determined by qPCR on an ABI QuantStudio 12K Flex (Applied Biosystems, Foster City, CA, USA) to ensure accurate loading. Cluster generation and sequencing were performed on an Illumina NovaSeq X Plus 25B platform (Illumina, San Diego, CA, USA) with paired-end chemistry, yielding 2 × 150 bp reads.

### 2.5. Bioinformatic Processing and Taxonomic Profiling

Raw FASTQ files underwent standard quality control to remove adapter contamination, low-quality reads, and reads containing > 5% ambiguous bases (N), generating high-quality clean reads. Clean reads were mapped to the human reference genome using BWA, and host-derived reads were removed to obtain effective microbial reads. Taxonomic assignment was performed with Kraken2 against the official minikraken2_v1_8GB database. Read counts assigned to taxa were used to estimate relative abundances. Annotations were aggregated to higher taxonomic ranks as required for downstream analyses.

### 2.6. Microbial Ecology Analyses

#### 2.6.1. Alpha Diversity

We computed richness indices (Chao1 and Abundance-based Coverage Estimator [ACE]) and diversity/evenness indices (Shannon, Simpson, and Pielou’s evenness). Group comparisons of diversity metrics were performed using the Kruskal–Wallis test. Plots and summary visualizations were generated with the CNSKnowall platform.

#### 2.6.2. Beta Diversity

Between-sample community dissimilarity was assessed by principal coordinate analysis (PCoA) based on Bray–Curtis distances. Group-level differences were tested by Permutational Multivariate Analysis of Variance (PERMANOVA), which partitions total variance using distance matrices and evaluates the explanatory power of grouping factors via permutation-based significance testing.

#### 2.6.3. Differential Abundance

Differential taxa were identified with Multivariate Association with Linear Models 2 (MaAsLin2) [20]. To ensure data robustness, only taxa with an average relative abundance greater than 0.0001% and a prevalence exceeding 10% across all samples were retained for downstream analyses. We considered genus-level differences significant at *p* < 0.05 and species-level differences at *q* < 0.20. For the differential species analysis, we adjusted for potential confounders of the gut microbiota—including the use of combination levodopa, MAOBI, primary dietary structure, smoking, and alcohol consumption—as covariates in a MaAsLin2 analysis (*p* < 0.05). All covariates were binary and derived from available metadata.

The selection of covariates for adjustment was guided by their documented association with the gut microbiota in the literature [21,22]. These variables were available only as binary categories (e.g., user/non-user). To address this limitation and to provide a more comprehensive perspective, we employed a dual analytical approach, presenting both unadjusted and covariate-adjusted results. The unadjusted results are presented as the main findings.

#### 2.6.4. Machine-Learning Validation

We tested whether different genera—identified as showing concordant directional changes in both PD vs. HC and DM vs. HC—could also discriminate PDDM from HC.

We compared transformed genus abundances across groups to determine whether PDDM showed the greatest enrichment. We compared three classifiers with default settings: support vector machine (RBF kernel), random forest, and XGBoost. Data were split 80/20 into training and test sets. On the training set, we performed stratified 5-fold cross-validation to select the primary model. The selected model was then evaluated on the held-out test set. To obtain a robust estimate, we additionally reported 5-fold cross-validated AUC on the full dataset. Performance metrics included area under the receiver operating characteristic curve (AUC-ROC). Predicted probabilities were calibrated using Platt scaling. SHAP (Tree Explainer) was used to quantify feature contributions. Genus names were italicized.

#### 2.6.5. Functional Profiling and GO–KO–KEGG Integration

Host-filtered metagenomic reads were used for open reading frame (ORF) prediction. Reads were mapped back to ORFs to obtain gene-level counts and transcripts per million (TPM), then annotated to Kyoto Encyclopedia of Genes and Genomes Orthology (KO) and Gene Ontology (GO). KO/GO-mapped genes were aggregated to KEGG pathways and GO categories. Differential testing (PD vs. HC, DM vs. HC) used Mann–Whitney U tests with Benjamini–Hochberg FDR correction. Effect sizes were summarized as log_2_(enrichment ratio, case/HC). GO terms and KEGG pathways were linked via shared KO identifiers to build the GO–KO–KEGG network [23,24,25]. Only bacterial KOs observed in the metagenomes were retained. Networks were visualized in Cytoscape (v.3.10.0) with node size proportional to degree (connectivity).

#### 2.6.6. Species–Pathway Correlations

Spearman’s rank correlations were computed between species abundances and KEGG metabolic pathways. For each species–pathway pair, we calculated the correlation coefficient (ρ) and *p* value. Associations meeting *p* < 0.05 and |ρ| > 0.30 were retained for downstream interpretation and visualization (heatmaps, Sankey diagrams, bubble plots) using Python (v.3.13.1) and R (v 4.3.3).

### 2.7. Statistical Analysis

All statistical analyses were conducted in SPSS 27.0. Categorical variables are presented as counts and percentages, and continuous variables as mean ± standard deviation. Between-group comparisons for categorical data used the chi-square test or rank-sum test as appropriate. For continuous data, independent-samples *t* tests were applied when the assumption of normality was met. Nonparametric tests (e.g., Mann–Whitney U) were used. Normality was assessed by the Kolmogorov–Smirnov test, and homogeneity of variance was evaluated to determine the suitability of parametric analyses. All tests were two-sided, with *p* < 0.05 considered statistically significant.

## 3. Results

### 3.1. Cohort Characteristics

A total of 324 participants were included. Table S1 summarizes the distribution of demographic variables (e.g., age, sex, BMI, years of education), FBG, and lifestyle/dietary factors (frequency of vegetable and meat intake, smoking, alcohol consumption, and coffee consumption) across the four groups. Except for FBG (*p* < 0.01), differences among the four groups were not statistically significant (all *p* > 0.05). In addition, comparisons between the PD and PDDM groups showed no significant differences in median UPDRS scores (28.5 vs. 34.5), UPDRS-III (17 vs. 21) scores, or median Hoehn–Yahr (H-Y) staging (both medians 2.0) (all *p* > 0.05), suggesting comparable disease severity. The proportions of participants using compound levodopa and monoamine oxidase type B inhibitors (MAOBIs) also did not differ between PD and PDDM (both *p* > 0.05). Detailed demographic and clinical characteristics for each group are provided in Table S1.

### 3.2. Overall Community Diversity: Convergent Trends Across Disease Groups

Analyses of gut microbiota diversity indicated that richness and evenness in the PD, DM, and PDDM groups were higher than those observed in the HC group. Pielou’s evenness index was significantly higher in PD and PDDM than in HC (*p* < 0.05), suggesting a more even distribution of taxa under disease states. Shannon diversity mirrored Pielou’s evenness index, and Simpson diversities further indicated a significant increase in α-diversity in PD, DM, and PDDM, collectively supporting a trend toward a more diversified community composition in disease. Detailed α-diversity metrics are provided in Table S2.

**Figure 1:**
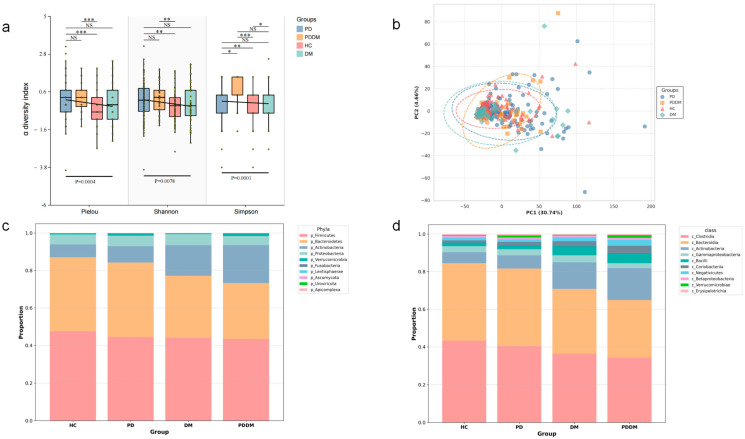
Global alterations in gut microbiome profiles across groups. (**a**) Boxplots comparing α-diversity indices (Pielou’s evenness, Shannon, Simpson). The y-axis shows z-score-standardized values. A fitted linear regression line is overlaid. * *p* < 0.05, ** *p* < 0.01, *** *p* < 0.001, NS, not significant. (**b**) Comparison of PCoA across groups. (**c**) Proportion of the top 10 taxa by mean relative abundance at the phylum level. (**d**) Proportion of the top 10 taxa by mean relative abundance at the class level.

The PCoA plot illustrates the differences in microbial community composition across groups (Figure 1b). Quantification by PERMANOVA showed that the separation between PDDM and HC was significant and the largest (R^2^ = 10.3%, *p* = 0.01), followed by DM and then PD, indicating that comorbidity might be associated with the most pronounced perturbation of the gut community (Table S3).

At high taxonomic ranks, PD and DM exhibited similar trends relative to HC. At the phylum level, Firmicutes and Bacteroidetes decreased, whereas Actinobacteria (significant in DM, *p* < 0.05) and Proteobacteria increased. At the class level, Clostridia and Bacteroidia were reduced in PD and DM compared with HC, while Actinobacteria and Gammaproteobacteria were elevated. The first three classes showed significant differences in DM versus HC (*p* < 0.05).

### 3.3. Shared Shifts in Gut Microbial Abundance at Genus and Species Levels Across Disease Groups

**Figure 2:**
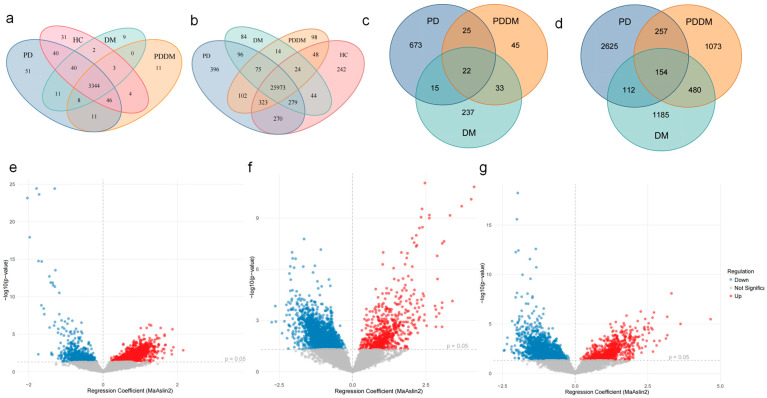
Genus- and species-level gut microbiome composition across groups (unadjusted model). (**a**) Genus-level Venn diagram displaying shared and unique genera among HC, PD, DM, and PDDM. (**b**) Species-level Venn diagram. (**c**,**d**) Venn diagrams of differentially abundant taxa identified by MaAsLin2 for each disease group versus HC. Intersection counts indicate taxa showing the same direction of change relative to HC (**c**, genus level; **d**, species level). Significance thresholds: (**c**) *p* < 0.05. (**d**) *q* < 0.20. (**e**–**g**) Volcano plots of species-level differential abundance for PD, DM, and PDDM versus HC, respectively. The x-axis shows MaAsLin2 regression coefficients, and the y-axis shows −log10(*q*), with *q* < 0.05 indicating significance.

We conducted a systematic analysis of the gut microbiota at the genus and species levels to resolve group-wise compositional features at a finer taxonomic resolution. Venn diagrams at both levels showed that the vast majority of genera and species were shared among HC, PD, DM, and PDDM, with only a small number of group-unique taxa (Figure 2a,b). Volcano plots further summarized species-level differences between each disease group and HC (Figure 2e–g, *p* < 0.05). Together, these results indicate that between-group differences were driven primarily by shifts in relative abundance within a shared set of taxa rather than by presence/absence. Accordingly, subsequent analyses focused on key taxa showing significant differential abundance across disease states.

Focusing on taxa that differed in all disease groups relative to HC, we identified 22 genera (Table S4) and 91 species that overlapped across PD, DM, and PDDM (Figure 2c,d). Importantly, the direction of change in PDDM mirrored that observed in PD and DM: taxa that increased (or decreased) in PD and DM showed the same directional shift in PDDM. We further filtered 17 species and valid binomial nomenclature (Table S5). After performing the covariate analysis, eight species remained significantly different *(p* < 0.05) (Table S6). These findings support a high degree of concordance in the dysbiosis trajectory between PD and DM and suggest that the comorbid state may reflect synergistic dysbiosis within the shared taxonomic core. To ensure biological relevance, taxa predominantly associated with environmental or food fermentation sources (e.g., *Marinomonas profundimaris*, *Lactobacillus cerevisiae*) were excluded from further interpretation.

**Figure A1:**
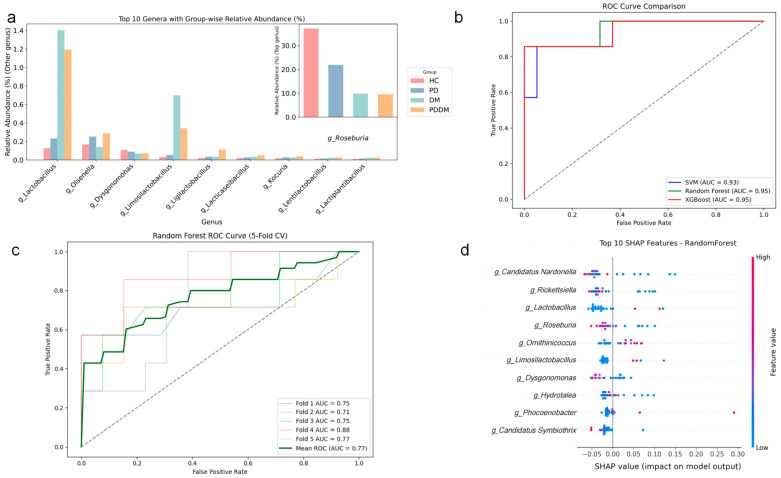
Genus panel and PDDM–HC classification performance. (**a**) Relative Abundance (%) of the Top 10 Genera Identified Among the 22 pre-specified genera across HC, PD, DM, and PDDM. (**b**) ROC curves on the held-out test set comparing SVM, random forest, and XGBoost. (**c**) Random-forest 5-fold cross-validated ROC curves with the mean ROC. (**d**) SHAP summary plot (top 10 genera).

Using the pre-specified 22-genus set, classifiers achieved moderate accuracy in distinguishing PDDM from HC. The random-forest model performed best among the three algorithms and was selected for further analysis. Across 5-fold cross-validation, mean AUC-ROC was 0.77. Predicted probabilities were reasonably calibrated after Platt scaling. SHAP analysis highlighted Candidatus *Nardonella*, *Roseburia*, *Ornithinicoccus*, *Lactobacillus*, *Limosilactobacillus* among the top contributors, consistent with genus-level abundance patterns (Figure A1).

### 3.4. Concordant GO Terms and KEGG Pathways Between PD and DM

**Figure 3:**
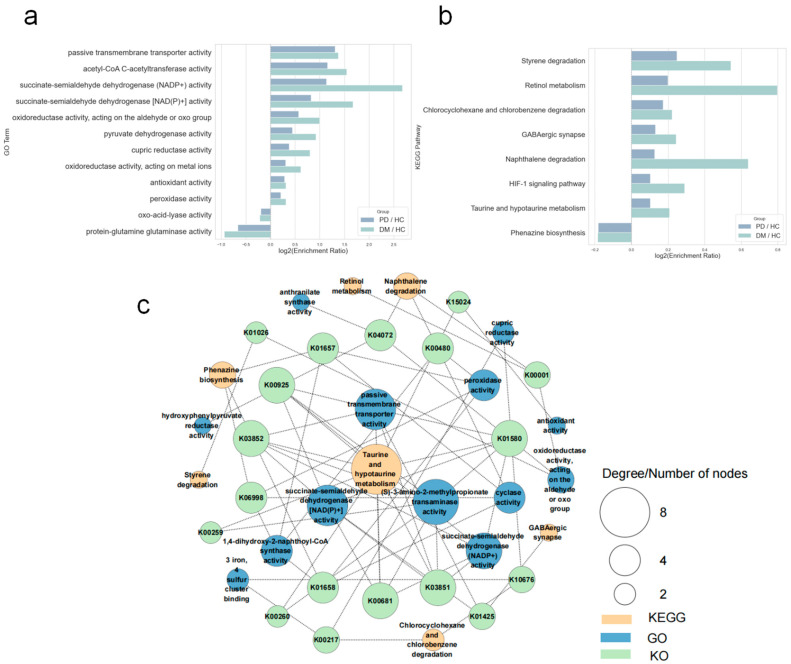
GO terms and KEGG pathways showing concordant changes in PD and DM. (**a**) GO terms with the same direction of change in PD/HC and DM/HC. The x-axis is log_2_(enrichment ratio, ER), defined as the ratio of enrichment strength in the case group to that in HC. log_2_(ER) > 0 indicates enrichment in cases, log_2_(ER) < 0 indicates depletion, and larger |log_2_(ER)| denotes stronger effects. (**b**) KEGG pathways with the same direction of change in PD/HC and DM/HC. Axis interpretation is as in (**a**). (**c**) GO–KO–KEGG interaction network. KO nodes (green) bridge their annotated GO terms (blue) and KEGG pathways (orange). Node size is proportional to degree, and edges represent annotation links. Central hub nodes highlight pathways that may regulate comorbidity-related metabolic and functional programs. Enrichment significance was controlled at FDR < 0.20.

To further probe shared functional signatures, we performed Gene Ontology (GO) and Kyoto Encyclopedia of Genes and Genomes (KEGG) enrichment analyses for PD/HC and DM/HC contrasts (FDR < 0.20). PD and DM showed the same direction of change for 24 GO terms and 8 KEGG pathways (Tables S7 and S8), most of which were enriched in the disease groups. We then linked GO terms to KEGG pathways through KEGG Orthology (KO) identifiers to construct a GO–KO–KEGG interaction network and to prioritize putative drivers or protectors of disease progression (Figure 3c). In this network, taurine and hypotaurine metabolism occupied a central position, while GO terms such as (S)-3-amino-2-methylpropionate transaminase activity, succinate-semialdehyde dehydrogenase [NAD(P)+] activity, and passive transmembrane transporter activity emerged as key functional nodes. Several KO enzymes acted as metabolic crossroads (participating in ≥2 biological processes), including K01580 (a transaminase implicated in taurine metabolism with both transamination and dehydrogenation roles), K04072 (naphthalene dioxygenase, catalyzing aromatic-ring hydroxylation and bridging aldehyde metabolism with aromatic compound degradation), K00217 (monooxygenase), and K00480 (quinone reductase), which contribute to specific or broad redox reactions. Collectively, these results indicate convergent dysregulation of oxidative stress handling, energy metabolism, xenobiotic degradation, and neuro-metabolic signaling in PD and DM, supporting a potential comorbidity-driving role of the gut microbiome. Notably, all KEGG pathways retained here were supported by observed KO expression in our metagenomes, minimizing confounding from homologous annotation.

### 3.5. Correlation Between Differentially Abundant Species and Metabolic Pathways

**Figure 4:**
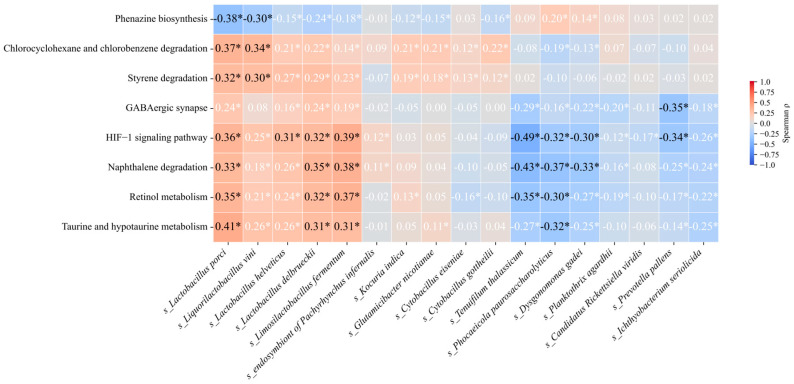
Spearman correlation heatmap between species and KEGG pathways. The heatmap displays correlations between 17 species (unadjusted model) and 8 key KEGG functional pathways. Cell values show the Spearman correlation coefficient (ρ), * *p* < 0.05.

We performed Spearman’s rank correlation analyses between the species that showed statistically significant and concordant changes across all three disease groups relative to HC and eight KEGG pathways that likewise met the significance threshold (Figure 4, Tables S9 and S10). Several species–pathway pairs exhibited moderate, significant correlations (|ρ| ≥ 0.30, *p* < 0.05) and warranted particular attention.

Notably, *Limosilactobacillus fermentum*, *Lactobacillus porci*, and *Lactobacillus delbrueckii* correlated positively with the HIF-1 signaling pathway, taurine and hypotaurine metabolism, naphthalene degradation, and retinol metabolism. In addition, *Lactobacillus porci* showed positive correlations with chlorocyclohexane and chlorobenzene degradation and styrene degradation, but a negative correlation with phenazine biosynthesis, suggesting a microbial subset with potential protective roles in disease. By contrast, *Phocaeicola paurosaccharolyticus*, *Prevotella pallens*, *Tenuifilum thalassicum*, and *Dysgonomonas gadei* were negatively correlated with the HIF-1 signaling pathway. Notably, *P. pallens* also showed a moderate negative correlation with the GABAergic synapse pathway. These associations indicate that taxa tightly linked to specific metabolic programs may hold functional relevance in disease mechanisms, requiring further investigation. At the same time, the correlation results for the group that included covariate analysis were relatively few. *Lachnospiraceae bacterium* OF09-6 showed positive correlations with naphthalene degradation and retinol metabolism.

In addition, we evaluated the associations of these species with UPDRS-III motor scores and FBG (Tables S11 and S12). The abundance of *L. porci* was positively correlated with both UPDRS-III scores (ρ = 0.229, *p* = 0.001) and FBG (ρ = 0.165, *p* = 0.006). Conversely, the abundance of *P. paurosaccharolyticus* was negatively correlated with both UPDRS-III scores (ρ = −0.148, *p* = 0.038) and FBG (ρ = −0.139, *p* = 0.021). The correlations for the remaining species (including species–clinical indicators after covariate analysis) with the clinical indicators were either inconsistent or not statistically significant.

### 3.6. Candidate Taxa Potentially Driving the Transition from PD to PDDM and Their Putative Pathways

To identify taxa that might drive the transition from a single disease (PD or DM) to PDDM, we compared PDDM with PD and with DM using MaAsLin2. No differentially abundant genera were found between PDDM and DM (all *q* > 0.6). At the species level (restricted to valid binomial Latin names), only *Sphingomonas melonis* (coef = −3.52) and *Nesterenkonia natronophila* (coef = −2.38) were significantly enriched in PD (*p* < 0.05).

**Figure 5:**
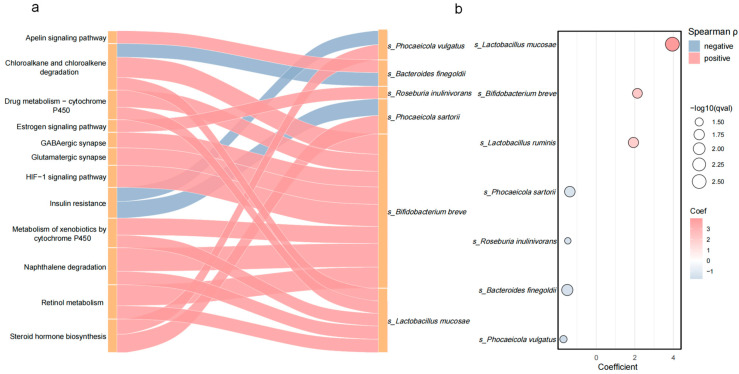
Differential species (unadjusted model) between PDDM and PD and their correlations with functional pathways. (**a**) Sankey diagram showing Spearman correlations between the differential species and KEGG functional pathways. Only associations with *p* < 0.05 and |ρ| > 0.3 are displayed. The width of the Sankey diagram’s bands represents the absolute value of Spearman’s correlation coefficient (|ρ|). (**b**) Bubble plot of species that differed significantly between PDDM and PD. “coef” denotes the MaAsLin2 regression coefficient (coef > 0, higher abundance in PDDM; coef < 0, lower abundance in PDDM).

We thus focused on the PDDM–PD contrast. To ensure robustness, we first identified species differentially abundant relative to HC, retaining seven shared between PDDM–HC and PD–HC comparisons. These were further assessed for associations with KEGG pathways (Spearman, *p* < 0.05, |ρ| > 0.30; Tables S12 and S13). The top 20 species (unadjusted model)–pathway associations are visualized in a Sankey diagram (Figure 5a), with abundance profiles in Figure 5b and Table S14.

In this unadjusted analysis, *Bifidobacterium breve* correlated with the most pathways (*n* = 13), showing positive links to naphthalene degradation, HIF-1 signaling, retinol metabolism, and chloroalkane/chloroalkene degradation and negative links to dioxin degradation and apelin signaling, suggesting pleiotropic metabolic roles. *Lactobacillus mucosae* and *L. ruminis* were positively associated with naphthalene degradation, retinol metabolism, and energy-related pathways, indicating roles in xenobiotic metabolism and oxidative stress handling. *Roseburia inulinivorans* showed bidirectional associations—positive with estrogen signaling and negative with naphthalene and retinol metabolism—implying involvement in endocrine and stress responses. Several taxa depleted in PDDM, including *Phocaeicola sartorii*, *P. vulgatus*, and *Bacteroides finegoldii*, were positively correlated with steroid hormone biosynthesis but negatively with insulin resistance, linking their loss to endocrine imbalance and metabolic risk.

In a complementary covariate-adjusted analysis, apart from the commonly identified differential taxa mentioned above (*L. mucosae* and *B. finegoldii*), *Bifidobacterium simiarum* was associated with the most pathways (*n* = 13), all positively. These pathways spanned neural signaling (GABAergic/glutamatergic synapses), HIF-1 signaling, and xenobiotic metabolism, suggesting broad regulatory roles in neurology and environmental stress. Among *Bacteroides* species, *B. acidifaciens*, *B. caecimuri*, and *B. pyogenes* consistently showed strong negative correlations with insulin resistance and positive correlations with steroid hormone biosynthesis, indicating a shared role in glucose and hormonal homeostasis. *B. pyogenes* additionally correlated negatively with HIF-1 signaling and xenobiotic degradation, reflecting broader functional associations. By contrast, *Butyricimonas vaginalis* was negatively correlated with multiple drug/xenobiotic metabolism pathways (e.g., HIF-1 signaling, insulin signaling, cytochrome P450 drug metabolism), consistent with a detrimental metabolic profile (Table S15).

## 4. Discussion

Multiple studies have documented the comorbidity of PD and DM and implicated shared mechanisms involving mitochondrial dysfunction, chronic systemic inflammation, and metabolic dysregulation [26,27,28]. In our consecutively enrolled PD cohort, the prevalence of DM was 22%, markedly higher than the 10·5% reported for the global population of comparable age (40–80 years) [2]. These observations raised the question of whether PD and DM share similar patterns of gut microbiota dysbiosis that, in turn, influence disease onset and progression.

We performed comparative shotgun metagenomic analysis in HC, PD, DM, and PDDM, systematically characterizing compositional features and functional abnormalities. We found that PD and DM exhibited convergent trends of gut microbial dysbiosis, each differing significantly from HC.

### 4.1. Concordant Gut Microbiota Dysbiosis in PD and DM

At the α-diversity level, we observed higher evenness and diversity in PD and PDDM compared to HC, suggesting a more diversified taxonomic composition in disease states.

The taxonomic shift, especially in Firmicutes and Bacteroidetes, was prominent in both PD and DM, consistent with prior reports by Takagi et al. and L. Mao et al. [29,30]. Despite extensive disease-specific alterations, PD and DM share a subset of taxa with consistent directional shifts. These overlapping signatures suggest partially convergent dysbiosis, which is further amplified in the comorbid state. Across the three disease groups, 22 genera and 91 species exhibited consistent directions of abundance change relative to HC. Among these, 13 genera showed the most pronounced alterations in PDDM. A diagnostic model built on these genera achieved an AUC of 0.77 in distinguishing PDDM from HC, suggesting that these taxa represent stable and representative features rather than random findings.

### 4.2. Common Mechanisms Revealed by Pathway Analysis

We explored shared functional signatures using GO and KEGG enrichment. We highlighted functions showing both strong effect sizes and high connectivity (Figure 3). These pathways support a model in which gut microbiome functional imbalance perturbs host immune, neural, and metabolic homeostasis, thereby fostering cross-disease pathology in PD–DM comorbidity.

#### 4.2.1. Taurine and Hypotaurine Metabolism as a Hub Pathway

This pathway was enriched in both diseases and showed the highest connectivity to differentially represented KO enzymes, suggesting a key role in disease-related metabolic perturbations. Taurine is a small molecule with anti-inflammatory and antioxidant properties that participates in diverse metabolic regulatory processes [31,32]. The biological actions of hypotaurine are closely coupled to its interconversion with taurine. Clinically, taurine supplementation in patients with T2DM already receiving metformin and DPP-4 inhibitors improved indices of glucose and fatty-acid metabolism [33]. Mechanistically, taurine has been reported to induce activation of FOXO1 and HNF4α, thereby promoting gluconeogenic programs [34], while also modulating pancreatic islet cell function [35]. In a PD MPTP mouse model, dysregulation of gut-microbiota-linked taurine metabolism was observed, and taurine administration prevented MPTP-induced motor deficits and dopaminergic neuronal loss [36]. Notably, in both disease contexts the “enrichment” we detected reflected microbial taurine/hypotaurine metabolic modules, whereas circulating levels of the corresponding host metabolites were reported to be reduced relative to controls [33,36]—a pattern consistent with enhanced microbial consumption or altered host–microbe exchange along this axis.

#### 4.2.2. Compensatory Activation of Detoxification Pathways

Xenobiotic degradation pathways, including naphthalene and chlorinated hydrocarbons, were also activated. This may reflect compensatory detoxification or chronic environmental exposures linked to mitochondrial dysfunction [37]. Experimental data have shown that plastics and pollutants disrupt energy metabolism, copper homeostasis, and lysosomal pathways, aggravating PD-like pathology [38,39,40]. Clearance of such xenobiotics can also drive activation of lipid-metabolic enzymes (e.g., acetyl-CoA transferases), and xenobiotic elimination pathways (cytochrome P450 systems) have been linked to abnormalities in glucose and insulin homeostasis [41,42].

Retinol metabolism was likewise enriched in both diseases. This pathway spans antioxidant defense, inflammation control, synaptic plasticity, and dopaminergic neuron survival and indirectly performs a detoxifying role in dopaminergic neurons [43]. Retinoid metabolites have also been reported to attenuate islet inflammation and reduce the risk of DM [44]. Finally, enrichment of the HIF-1 signaling pathway in the disease groups likely reflects a compensatory response to metabolic stress, oxidative pressure, or environmental toxicants [45].

Our findings support a unified model of gut-microbiome-driven comorbidity in PDDM. We propose that the synchronous dysregulation of detoxification, redox homeostasis, and stress signaling creates a self-reinforcing cycle. In this model, an impaired microbial detoxification capacity increases the host’s vulnerability to environmental insults; a disrupted taurine-metabolizing niche depletes a key reservoir for antioxidant and neuroprotective defenses; and persistent HIF-1 signaling sustains a pro-inflammatory milieu. This triad of functional impairments converges to generate a systemic background that potently accelerates both metabolic dysfunction and neurodegeneration.

### 4.3. Key Genus Markers Indicated by Taxon–Function Correlations

#### 4.3.1. Shared Species (PD and DM)

Correlation analyses identified taxa tightly associated with these pathways. *Limosilactobacillus fermentum*, *Lactobacillus porci*, and *Lactobacillus delbrueckii* were generally increased in all disease groups and were significantly associated with multiple functional pathways, suggesting a modulatory role under comorbid conditions. *L. fermentum* U-21 secretes an ATPase chaperone with antioxidative activity that helps maintain intracellular protein structural stability [46], improves motor coordination, and prevents degeneration of dopaminergic neurons in PD models [47]. Extensive studies support protective roles of *lactobacilli* in PD and T2D models [48,49]. *Lactobacilli* can produce γ-aminobutyric acid (GABA) [50]. They can also activate retinol metabolism pathways and regulate mucosal immune responses [51]. In addition, several *Lactobacillus* spp. promote detoxification by upregulating host detoxifying enzymes and binding harmful compounds [52]. Consistent with these reports, *L. porci* and *L. delbrueckii* in our dataset showed moderate positive correlations with the aforementioned pathways. After covariate adjustment, the significance of *L. porci* persisted, while *Lachnospiraceae bacterium* OF09-6 was positively associated with naphthalene degradation and retinol metabolism—a pattern in line with the metabolic roles of other butyrate-producing Lachnospiraceae that support gut barrier and immune functions [53].

#### 4.3.2. Conversion Species (PD → PDDM)

In the unadjusted analysis, *Bifidobacterium breve*, further enriched in PDDM, correlated with the largest number of pathways, including xenobiotic degradation and antioxidant signaling. *B. breve* has been reported to reduce intestinal lipid uptake and blood cholesterol. In combination with berberine, it improved metabolic status in patients with T2DM [54]. *B. breve* also produces indole-3-lactic acid, which has shown therapeutic potential by reducing neuroinflammation [55]. Among taxa with decreased abundance, *Phocaeicola vulgatus* was reported to alleviate diet-induced metabolic dysfunction via 3-hydroxyphenylacetic acid-mediated downregulation of histone acetylation [56]. Another species, *Lactobacillus mucosae*, through its exopolysaccharides and secretome, modulated the host bile-acid pool and immune responses, with applications reported in metabolic and neurological disorders [57,58].

With covariate adjustment, *B. simiarum* replaced *B. breve* as the bifidobacterial marker and showed uniformly positive associations across neural signaling (GABAergic/glutamatergic), HIF-1 signaling, and xenobiotic metabolism. These associations may reflect genus-conserved functions described for other *Bifidobacterium* species [59]; however, species-specific mechanisms for *B. simiarum* remain to be established. Within *Bacteroides*, *B. acidifaciens*, *B. caecimuri*, and *B. pyogenes* consistently exhibited negative correlations with insulin resistance and positive correlations with steroid hormone biosynthesis. *B. acidifaciens* has been shown in mice to reduce weight gain and adiposity through activation of peroxisome proliferator-activated receptor alpha (PPARα) and enhancement of GLP-1 signaling, accompanied by reduced intestinal DPP-4 activity [60]. By contrast, *Butyricimonas vaginalis* showed negative correlations with multiple drug/xenobiotic pathways (including HIF-1 and insulin signaling; cytochrome-P450 drug metabolism), consistent with a detrimental metabolic profile (Table S15). *B. vaginalis* has been implicated in the disruption of gut epithelial integrity and in increased inflammatory and oxidative responses (elevated serum interleukin-1β, myeloperoxidase, and cyclooxygenase-2) [61], which could exacerbate mucosal vulnerability in PD and heighten the risk of comorbid progression.

This study has several limitations. First, as a cross-sectional analysis, it cannot establish causality. Second, functional inference was based on metagenomic annotations without metabolomic validation. Third, although we adjusted for key covariates, residual confounding from unmeasured medications (e.g., metformin), more granular dietary components, and sex cannot be excluded. Furthermore, the enrichment of taxa traditionally considered “beneficial” (e.g., *lactobacilli*, *B. breve*) in disease groups highlights the context-dependent nature of microbial functions, which may reflect strain-level heterogeneity, disease-specific adaptations, or compensatory responses. Future work should employ longitudinal, strain-resolved, and multi-omics approaches to validate these candidate taxa and pathways and clarify their causal roles.

## 5. Conclusions

In summary, PD and DM exhibited concordant taxonomic and functional dysbiosis, with comorbidity amplifying these perturbations. Shared enrichment of taurine/hypotaurine metabolism, detoxification pathways, retinol signaling, and HIF-1 signaling, along with consistent taxon–function correlations, point to common microbiome-mediated mechanisms underlying comorbidity. These findings position the shared gut microbial signature as a scientific foundation for future research into risk stratification and mechanism-driven interventions for PD–DM comorbidity.

## Data Availability

The data supporting this study are available in the article and its Supplementary Information. Relevant datasets have been deposited in the National Microbiology Data Center (NMDC) under accessions NMDC20397890, NMDC40085044, NMDC20400380 and NMDC20401215.

## Supplementary Materials

The following supporting information can be downloaded at https://www.mdpi.com/article/10.3390/microorganisms13122705/s1: Table S1: Demographic characteristics of participants by group. Table S2: Alpha-diversity comparison among groups. Table S3: PERMANOVA among Groups. Table S4: Shared taxa among PD, DM, and PDDM (genus level). Table S5: Shared taxa among PD, DM, and PDDM (species level). Table S6: Shared species between PD, DM, and PDDM groups and their abundance (adjusted model). Table S7: GO term enrichment results across groups. Table S8: KEGG pathway enrichment results across groups. Table S9: Spearman correlation results for the commonly significant differential species and KEGG pathways between the three disease groups and HC. Table S10: Spearman correlation results for the significant differential species and blood glucose and clinical-scale data. Table S11: Spearman correlation results for the covariate-adjusted differential species (MaAsLin2) and blood glucose and clinical-scale data. Table S12: Spearman correlation results for the significant differential species and KEGG pathways between PD and PDDM. Table S13: Spearman correlation results for the covariate-adjusted differential species (MaAsLin2) and KEGG pathways between PD and PDDM. Table S14: Differential gut microbial species between PDDM and PD. Table S15: Differential gut microbial species between PDDM and PD (adjusted model).

## Abbreviations

The following abbreviations are used in this manuscript:

ACE	Abundance-based Coverage Estimator
AUC-ROC	area under the receiver operating characteristic curve
BMI	body mass index
BWA	Burrows–Wheeler Aligner
D_2_R	dopamine D2 receptor
DAT	dopamine transporter
DM	diabetes mellitus
DNA	deoxyribonucleic acid
DPP-4	dipeptidyl peptidase-4
FASTQ	raw sequencing read file format
GABA	γ-aminobutyric acid
GLP-1	glucagon-like peptide-1
GO	Gene Ontology
HC	healthy controls
HIF-1	hypoxia-inducible factor-1
HR	hazard ratio
IAPP	islet amyloid polypeptide
InsR	insulin receptor
IRR	incidence rate ratio
KEGG	Kyoto Encyclopedia of Genes and Genomes
KO	KEGG Orthology
MaAsLin2	Multivariate Association with Linear Models 2
MAPK	mitogen-activated protein kinase
MDS	Movement Disorder Society
MOABI	monoamine oxidase type B inhibitors
OR	odds ratio
ORF	open reading frame
PCoA	principal coordinates analysis
PD	Parkinson’s disease
PDDM	PD with type 2 diabetes
PERMANOVA	Permutational Multivariate Analysis of Variance
PPARα	peroxisome proliferator-activated receptor alpha
RBF	radial basis function
SHAP	SHapley Additive exPlanations
SVM	support vector machine
T2DM	type 2 diabetes mellitus
TMAO	trimethylamine N-oxide
TPM	transcripts per million
UPDRS	Unified Parkinson’s Disease Rating Scale
XGBoost	eXtreme Gradient Boosting
